# Infarto com Supra-ST em Adulto Jovem: Rara Apresentação de Mixoma Atrial Gigante

**DOI:** 10.36660/abc.20230538

**Published:** 2024-04-04

**Authors:** Rodrigo Rufino Pereira Silva, Carolina Jerônimo Magalhães, Rafael Silvestre Vieira da Silva, Giulia Antoni Ferreira Rocha, Paulo Ernando Ferraz Cavalcanti, Sérgio Tavares Montenegro

**Affiliations:** 1 Pronto Socorro Cardiológico de Pernambuco Professor Luiz Tavares Recife PE Brasil Pronto Socorro Cardiológico de Pernambuco Professor Luiz Tavares (PROCAPE), Recife, PE – Brasil; 2 Universidade de Pernambuco Recife PE Brasil Universidade de Pernambuco (UPE), Recife, PE – Brasil

**Keywords:** Infarto do Miocárdio com Supradesnivelamento do Segmento ST, Adulto Jovem, Mixoma, Diagnóstico por Imagem/métodos

## Abstract

Neoplasias cardíacas são raras, tendo como principal representante o mixoma atrial (MA), que corresponde a cerca de metade de todos os casos. O MA tem incidência estimada entre 0.001% e 0.3% na população em geral, no entanto apenas aproximadamente 0,06% desses cursam com eventos embólicos coronarianos.

Homem de 33 anos, tabagista, admitido com quadro de precordialgia intensa e irradiação para membro superior esquerdo com duração de uma hora. O eletrocardiograma evidenciou elevação de segmento ST nas derivações D2, D3 e aVF troponina sérica elevada, confirmando infarto com supra desnivelamento do segmento ST (IAMCSST). Foi realizada cineangiocoronariografia, a qual revelou oclusão em terço proximal de artéria coronária direita por trombo. Realizada tentativa de aspiração do trombo, sem sucesso, seguido por angioplastia primária com balão sem colocação de stent. Durante a investigação do quadro, paciente realizou ecocardiograma transtorácico o qual demonstrou massa homogênea de superfície regular, de 5.2 cm x 2.3 cm, aderida ao septo interatrial, com lobulações de características emboligênicas prolapsando para valva mitral e ventrículo esquerdo na diástole, compatível com MA. Foi realizada ressecção cirúrgica com paciente evoluindo assintomático, recebendo alta para seguimento ambulatorial.

O caso relatado difere em idade e sexo do perfil epidemiológico típico sendo um dos poucos descritos com acometimento da parede inferior apresentando a artéria coronária direita como culpada. Este relato ratifica a importância do diagnóstico diferencial frente às apresentações de IAMCSST em jovens.

## Introdução

Tumores cardíacos são entidades raras quando considerada a totalidade dos casos, apresentando prevalência aproximada de 0,001% a 0,3%^1^ na população geral. A maior parte dos tumores cardíacos é de etiologia secundária, sendo representados pelas metástases. Dentre os tumores primários, mais incomuns, aproximadamente 80% são benignos, sendo seu principal representante o mixoma atrial (MA). O MA pode se originar em qualquer câmara cardíaca, mas aparece no átrio esquerdo em mais de 75% dos casos.^1 , 2^

Embora biologicamente benigno, o MA tem potencial danoso associado à sua capacidade tromboembólica e/ou obstrutiva. Tromboembolismo por MA é uma complicação reconhecida e relativamente comum, podendo ocorrer em até 40% dos casos. Quando ocorre, geralmente afeta o sistema nervoso central, sendo causa potencial de acidente vascular encefálico.³ Mais raramente, pode haver embolia coronariana, resultando em infarto agudo do miocárdio (IAM) em até 0,06% dos casos.^4^ Relatamos a seguir uma rara apresentação de MA em paciente adulto jovem: IAM de parede inferior secundário a processo de tromboembolismo coronariano.

## Relato de Caso

**Figura 1:**
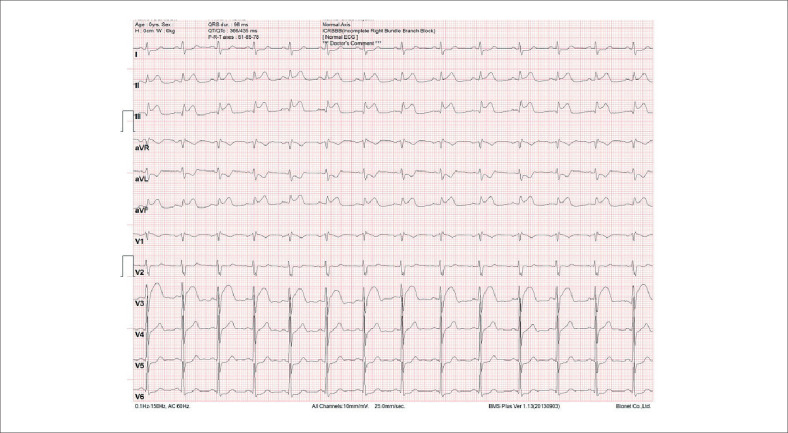
Eletrocardiograma admissional de 12 derivações evidenciando supradesnivelamento do segmento ST em derivações inferiores: D2, D3 e aVF e infradesnivelamento do segmento ST em parede lateral (D1 e aVL).

Paciente de 33 anos, masculino, tabagista ativo (carga tabágica estimada em 40 maços-ano), previamente hígido, foi admitido em emergência cardiológica de serviço terciário de cardiologia em Recife-PE com quadro de precordialgia intensa com irradiação para membro superior esquerdo de início uma hora antes da admissão, sem outros sintomas associados. O paciente negava comorbidades conhecidas e uso de quaisquer medicações ou ilícitos. Sinais vitais e exame físico admissionais sem alterações. Na emergência, foi realizado eletrocardiograma ( Figura 1 ) o qual evidenciava elevação de segmento ST nas derivações D2, D3 e aVF, com valor de troponina sérica de 20.350,00 ng/dL (valor de referência <40 ng/dL), confirmando diagnóstico de IAM com supradesnivelamento do segmento ST (IAMCSSST) de parede inferior. Nesse momento, foi realizada dose de ataque com aspirina e clopidogrel.

**Figura 2:**
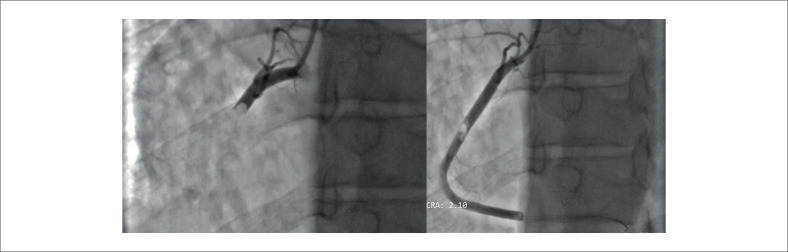
Cineangiocoronariografia com oclusão em ⅓ proximal de ACD com imagem negativa sugestiva de trombo à esquerda, e, à direita, imagem de migração distal do trombo após tentativa de recanalização.

Posteriormente, o paciente seguiu para cineangiocoronariografia de emergência a qual revelou oclusão em terço proximal de artéria coronária direita (ACD) com imagens negativas na luminografia, sugestivas de oclusão por trombo ( Figura 2 ). Nesse momento, foi realizada aspiração do trombo, sem sucesso. A seguir, deu-se seguimento à angioplastia primária com balão sem colocação de stent. Após angioplastia houve embolização distal com oclusão do ramo ventricular posterior. Nesse contexto, foi administrado tirofiban. Paciente evoluiu hemodinamicamente estável, sem novos episódios de dor e sem intercorrências.

Após a alta da unidade de terapia intensiva, ainda durante o internamento e para fins de investigação do quadro, o paciente realizou ecocardiograma transtorácico. O exame revelou uma massa homogênea de superfície regular em átrio esquerdo, medindo 5.2 cm x 2.3 cm, aderida ao septo interatrial, com lobulações de características emboligênicas prolapsando para valva mitral e ventrículo esquerdo na diástole, sugestivo de MA. Havia, nesse momento, acinesia de parede inferior e fração de ejeção pelo método de Simpson de 45%, sem outras alterações.

**Figura 3:**
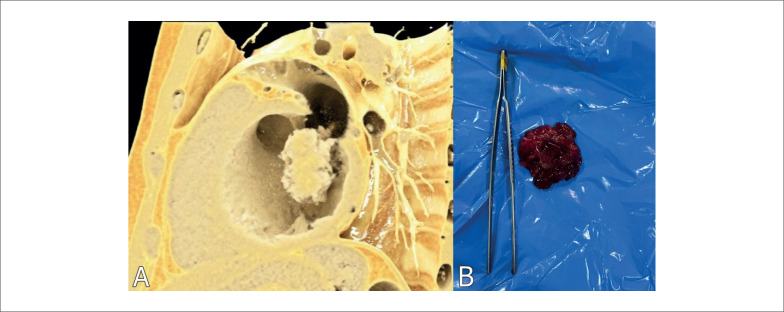
A: Reconstrução com renderização em 3D de angiotomografia evidenciando massa volumosa e multilobulada em átrio esquerdo com invasão para ventrículo esquerdo e folheto anterior de válvula mitral; B: Peça cirúrgica do mixoma atrial.

Como paciente era tabagista e apresentou quadro de síndrome coronariana aguda com alteração segmentar ao ecocardiograma, foi realizada angiotomografia de coronárias com score de cálcio e reconstrução em 3D para descartar a possibilidade de doença coronariana com possíveis lesões instáveis menores como etiologia do IAM ( Figura 3 ). Além disso, a imagem auxiliaria na avaliação do grau de invasividade ventricular da massa e o acometimento da valva mitral, dado que contribuiria para programação cirúrgica pela possibilidade de troca valvar.

Não foram evidenciadas lesões ateroscleróticas ao método, ratificando a causa tromboembólica do IAMCSSST. Assim, foi realizada ressecção cirúrgica do MA, sem troca valvar e sem intercorrências. O paciente recebeu alta hospitalar no quinto dia de pós-operatório com bom estado geral e assintomático, com programação de acompanhamento ambulatorial.

A peça cirúrgica ( Figura 3 ) foi enviada para estudo histopatológico, o qual revelou material amorfo envolto em estroma mixomatoso composto por endotélio e células poligonais/estreladas, mal organizadas, com alterações degenerativas e sem outros processos malignos associados. Os achados foram compatíveis com MA.

## Discussão

Embora o risco de embolização sistêmica pelo MA exista, sua ocorrência em território coronariano é extremamente rara, estimando-se incidência aproximada de 0,06% em todos os casos.^4^ A baixa ocorrência de embolia para as artérias coronarianas pode ser explicada pela sua angulação em relação à aorta, somada à proteção da válvula aórtica durante a sístole e do reduzido diâmetro dessas artérias.^5^ Dentre os IAMs causados por MA, a maior parte dos relatos na literatura descreve casos de IAM sem supradesnivelamento do segmento ST e, quando IAMCSSST, o acometimento da artéria descendente anterior é o mais comum, possivelmente por motivos anatômicos.

Existem na literatura 18 artigos do tipo relato de caso que reportam IAMCSST decorrente de embolia por MA esquerdo. Dentre 18 pacientes, 8 eram homens e 10 mulheres. Houve acometimento de ACD apenas em 2 casos, sendo os dois pacientes do sexo masculino.

Não houve complementação de estudo com angiotomografia de coronárias em nenhum deles. Entre todos os casos de MA, o perfil típico descrito é de mulheres, incidência de 3:1, entre a quarta e sétima décadas de vida, ocorrendo de forma esporádica.^6^

No compilado mais recente sobre o tema da revisão de Al Zahrani et al.,^7^ foram estudados 17 casos relatados em língua inglesa entre 2003 e 2014. Nesta série de casos, dez dos 17 casos (59%) apresentavam angiografia de coronárias normais. Dentre esses, 70% tinham menos de 45 anos. A razão para coronariografias normais em pacientes com IAM por complicação de MA ainda não é claramente compreendida. A recanalização espontânea após embolização do MA ou a demora em realizar o exame são sugeridos como causas prováveis,^7^ porém isso difere do caso relatado ( Figura 2 ).

Na maior parte das vezes, não é recomendada a aspiração de trombos em cineangiocoronariografia de rotina. A trombectomia é uma alternativa possível, mas carece de validação na literatura com mais estudos.^8^ No entanto, no contexto de embolização trombogênica secundária à processos tumorais como MA, essa abordagem já foi descrita com sucesso e foi a utilizada no caso descrito.^9 , 10^

A ressecção cirúrgica completa é a única terapia efetiva para alterar o prognóstico com tratamento curativo. Destaca-se a programação do pré-operatório a fim de planejamentos embasados e da diminuição de complicações inesperadas, o qual foi realizado no caso com exames de imagem.

Para fechamento do defeito cirúrgico pós excisional do MA, podem ser usados patch de pericárdio ou Dacron. No intraoperatório é essencial, para reduzir o risco de fragmentação e possíveis novos episódios de embolização, evitar palpação vigorosa ou manipulação da massa, devendo estas serem realizadas apenas após cardioplegia pela friabilidade do tecido.^9 , 10^ Para os pacientes abordados com ressecção completa, a sobrevida em longo prazo é excelente e a recorrência é rara. O risco geral de recorrência é entre 12% e 22% para mixoma familiar e complexo, respectivamente.

Para tumores esporádicos é de apenas 1 a 3%, como o caso do paciente relatado. Acompanhamento regular com ecocardiografia é indicado em todos os casos.^9 , 10^

Este caso ressalta uma causa rara de IAM e a importância em explorar possíveis diagnósticos diferenciais para casos de síndrome coronariana aguda em pacientes jovens sem fatores de risco para doença aterosclerótica. Denota-se A necessidade de tratamento cirúrgico definitivo em casos de MA como melhor possibilidade terapêutica. A celeridade da estratégia invasiva precoce, quando indicada, é fundamental para o sucesso na maior parte dos casos.

## Conclusão

Relatado raro caso de embolização coronariana por MA gigante, o qual se apresentou como IAMCSST em paciente jovem. Além do quadro infrequente, o perfil epidemiológico do paciente – homem jovem – e a artéria coronariana acometida – ACD – torna o caso ainda mais singular. Ademais, ressalta-se a importância do diagnóstico diferencial de patologia de apresentações incomuns de doenças prevalentes no dia a dia do cardiologista, como no caso do IAMCSST.
