# Supplementary material for: Infarto com Supra-ST em Adulto Jovem: Rara Apresentação de Mixoma Atrial Gigante
Source: Arq Bras Cardiol. 2024 Apr 4;121(3):e20230538. [Article in Portuguese] doi: 10.36660/abc.20230538 (PMC11081103; doi:10.36660/abc.20230538)
Supplement: Translation

# Translation: Acute ST-Elevation Myocardial Infarction in a Young Adult: Rare Presentation of Giant Atrial Myxoma

**DOI:** 10.36660/abc.20230538i

**Authors:** Rodrigo Rufino Pereira Silva, Carolina Jerônimo Magalhães, Rafael Silvestre Vieira da Silva, Giulia Antoni Ferreira Rocha, Paulo Ernando Ferraz Cavalcanti, Sérgio Tavares Montenegro

**Affiliations:** 1 Recife PE Brazil Pronto Socorro Cardiológico de Pernambuco Professor Luiz Tavares (PROCAPE), Recife, PE – Brazil; 2 Recife PE Brazil Universidade de Pernambuco (UPE), Recife, PE – Brazil

**Keywords:** ST Elevation Myocardial Infarction, Young Adult, Myxoma, Diagnostic Imaging/methods

## Abstract

Cardiac tumors are rare entities, among which atrial myxoma (AM) stands as the most frequent, accounting for approximately half of all reported cases. The incidence of AM is estimated to range from 0.001% to 0.3% within the general population, yet only about 0.06% of these cases present with coronary embolic events.

We report on a 33-year-old male smoker who experienced acute, severe precordial pain radiating to the left upper limb, lasting for one hour. The electrocardiographic evaluation demonstrated ST-segment elevation in leads D2, D3, and aVF, alongside significantly elevated serum troponin levels, confirming a diagnosis of ST-segment elevation myocardial infarction (STEMI). Subsequent coronary angiography revealed proximal occlusion of the right coronary artery due to thrombus. An initial attempt of thrombus aspiration was unsuccessful, followed by primary angioplasty with balloon inflation without stent placement. Further diagnostic exploration through transthoracic echocardiography identified a homogenous, smooth-surfaced mass measuring 5.2 cm x 2.3 cm attached to the interatrial septum. This mass, characterized by lobulations, prolapsed into the mitral valve and left ventricle during diastole, consistent with AM. Surgical resection of the mass was successfully performed, with the patient being discharged asymptomatic.

In the reported case, the patient's profile, notably his age, and gender, diverges from the typical epidemiological characteristics associated with AM. This case adds to the limited number of reports where the inferior wall is affected by the right coronary artery being occluded. This report emphasizes the significance of differential diagnoses in younger patients presenting with STEMI.

## Introduction

Cardiac tumors are rare entities, exhibiting a prevalence of approximately 0.001% to 0.3% in the general population.^1^The majority of cardiac tumors have secondary etiology, represented by metastases. Among primary tumors, which are more uncommon, about 80% are benign, with atrial myxoma (AM) being the most prominent representative. AM can originate in any cardiac chamber, but in over 75% of cases is found in the left atrium.^1 , 2^

Although biologically benign, AM carries harmful potential due to its thromboembolic and/or obstructive capacity. Thromboembolism from AM is a recognized and relatively common complication, occurring in up to 40% of cases. When it occurs, it more commonly affects the central nervous system, potentially leading to strokes.³ Additionally, but more rarely, coronary embolism can occur and result in myocardial infarction (MI) in up to 0.06% of cases.^4^

We report a rare presentation of AM in a young adult patient: inferior wall ST-segment elevation MI (STEMI) secondary to coronary thromboembolism.

## Case Report

A previously healthy 33-year-old male patient was admitted to the cardiac emergency department of a tertiary cardiology service, presenting with intense precordial pain radiating to the left upper limb starting one hour prior to admission. He had no other associated symptoms. The patient was an active smoker (estimated tobacco load of 40 pack-years), had no known comorbidities, and denied the use of any medications or narcotics. Vital signs and physical examination were unremarkable. In the emergency department, an electrocardiogram (ECG) ( Figure 1 ) was performed, which showed ST-segment elevation in leads D2, D3, and aVF. Additionally, serum troponin levels were 20,350.00 ng/dL (reference value <40 ng/dL), confirming the diagnosis of inferior wall STEMI. At this point, aspirin and clopidogrel were administered.

**Figure 1:** 12-lead admission ECG showing ST-segment elevation in inferior leads: D2, D3, and aVF and ST-segment depression in the lateral wall (D1 and aVL).

Subsequently, the patient underwent emergency coronary angiography, which revealed a proximal occlusion of the right coronary artery (RCA) with negative images on luminescence, suggesting thrombotic occlusion ( Figure 2 ). Thrombus aspiration was unsuccessfully attempted. Furthermore, primary balloon angioplasty was performed without stent placement. Post-angioplasty, there was distal embolization with occlusion of the posterior ventricular artery, and tirofiban was administered. The patient was hemodynamically stable, without new episodes of pain or complications.

**Figure 2:** Coronary angiography showing proximal occlusion of the RCA with a negative image suggestive of thrombus on the left, and on the right, an image of distal thrombus migration after recanalization attempt.

After discharge from the intensive care unit, the patient underwent a transthoracic echocardiogram. It revealed a homogeneous mass with a regular surface in the left atrium, measuring 5.2 cm x 2.3 cm, adhered to the interatrial septum, with emboligenic lobulations prolapsing toward the mitral valve and left ventricle during diastole, suggestive of an AM. The echocardiogram additionally revealed inferior wall akinesia and an ejection fraction by the Simpson method of 45%; no other alterations were reported.

A coronary CT angiography with calcium scoring and 3D reconstruction was performed to rule out the possibility of coronary artery disease with minor unstable lesions ( Figure 3 ) since the patient smoked and presented with an acute coronary syndrome evolving to ventricular segmental dysfunction. Moreover, the imaging helped to assess the degree of ventricular invasiveness of the mass and the involvement of the mitral valve, contributing to surgical planning ofpotential valve replacement.

**Figure 3:** A: 3D rendering reconstruction of angiotomography highlighting a voluminous and multilobulated mass in the left atrium with invasion into the left ventricle and the anterior leaflet of the mitral valve; B: Surgical specimen of the atrial myxoma.

No atherosclerotic lesions were identified, and the calcium score was zero, reaffirming the thromboembolic STEMI. Therefore, AM surgical resection ( Figure 3 ) was performed without valve replacement and complications. The patient was discharged on the fifth postoperative day, asymptomatic.

Histopathological specimen study revealed amorphous material surrounded by a myxomatous stroma composed of endothelium and polygonal/stellate cells, poorly organized, with degenerative changes and no other associated malignant processes. These findings were consistent with an AM.

## Discussion

Although the risk of systemic embolization by AM exists, its occurrence in the coronary territory is exceedingly rare, with an estimated incidence of approximately 0.06%.^4^Their angulation may explain the infrequent embolization of coronary arteries to the aorta, combined with the protection afforded by the aortic valve during systole and the small diameter of these arteries.^5^Nonetheless, among MI caused by AM, there are more case reports in the literature of non-ST-elevation MI, and, in cases of STEMI, the involvement of the anterior descending artery is the most common, likely due to anatomical reasons.

A literature review returns only 18 case report articles of STEMI due to left AM embolism. Among these 18 patients, 8 were men and 10 were women. Only 2 cases involved the RCA, and both patients were male. None of them had complemented the study with coronary CT angiography. Among all these AM cases, the typical profile described was: women – incidence ratio of 3:1 – between the fourth and seventh decades of life.^6^

In the most recent compilation on the topic, Al Zahrani et al.^7^studied 17 cases reported in English between 2003 and 2014. In this case series, ten of the 17 cases (59%) presented with normal coronary angiography. Among these, 70% were under 45 years old. The reason for normal coronary angiographies in patients with MI due to AM complications is not yet clearly understood. Spontaneous recanalization after AM embolization or delay in performing the exam are suggested as probable causes,^7^yet this differs from the case reported ( Figure 2 ).

Generally, thrombus aspiration in routine coronary angiography is not recommended. Thrombectomy is a possible alternative, but it lacks validation in the literature with further studies.^8^However, in the context of thrombogenic embolization secondary to tumoral processes like AM, this approach has been successfully described and was utilized in the reported case.^9 , 10^

Complete surgical resection is the only effective therapy to alter the prognosis with curative treatment. Pre-operative planning is emphasized to enable well-founded plans and decrease unexpected complications, as was performed in the case using imaging tests.

For the closure of the post-excisional surgical defect in the interatrial septum, pericardial or Dacron patches can be used. Intraoperatively, to reduce the risk of fragmentation and potential new embolization episodes, vigorous manipulation of the mass should be avoided and should only be performed after cardioplegia due to the tissue's friability.^9 , 10^For patients undergoing complete resection, long-term survival is excellent, and recurrence is rare. The overall risk of recurrence is between 12% and 22% for familial and complex myxoma, respectively. For sporadic tumors, it is only 1 to 3%, as in the reported patient's case. Regular follow-up with echocardiography is indicated in all cases.^9 , 10^

This report highlights a rare cause of MI and the importance of exploring potential differential diagnoses for acute coronary syndrome in young patients without atherosclerotic risk factors. This case also denotes the importance of definitive surgical treatment in cases of AM as the best therapeutic possibility. The early invasive strategy, when indicated, is fundamental to success.

## Conclusion

We reported the rare case of coronary embolization by a giant AM, which presented as STEMI in a young patient. Beyond the infrequent presentation, the epidemiological profile of the patient – a young man – and the affected coronary artery – RCA – makes this case even more unique. Furthermore, the reported case underscores the importance of differential diagnosis in a scenario as common to cardiologists as MI.
